# Neuroimaging in the Kleine-Levin Syndrome

**DOI:** 10.1007/s11910-018-0866-y

**Published:** 2018-07-21

**Authors:** Maria Engström, Francesco Latini, Anne-Marie Landtblom

**Affiliations:** 1 Department of Medicine and Health Sciences, Linköping University, Linköping, Sweden; 2 Center for Medical Image Science and Visualization (CMIV), Linköping University, Linköping, Sweden; 3 CMIV, Linköpings universitet/US, 581 83 Linköping, Sweden; 4 Department of Neuroscience, Section of Neurosurgery, Uppsala University, Uppsala, Sweden; 5 Department of Neuroscience, Section of Neurology, Uppsala University, Uppsala, Sweden

**Keywords:** Kleine-Levin syndrome (KLS), Functional magnetic resonance imaging (fMRI), Positron emission tomography (PET), Single photon emission computed tomography (SPECT), Magnetic resonance spectroscopy (MRS), Diffusion weighted imaging (DWI)

## Abstract

**Purpose of Review:**

The purpose was to review the most recent literature on neuroimaging in the Kleine-Levin syndrome (KLS). We aimed to investigate if frontotemporal and thalamic dysfunction are key KLS signatures, and if recent research indicates other brain networks of interest that elucidate KLS symptomatology and aetiology.

**Recent Findings:**

In a comprehensive literature search, we found 12 original articles published 2013–2018. Most studies report deviations related to cerebral perfusion, glucose metabolism, or blood-oxygen-level-dependent responses in frontotemporal areas and/or the thalamus. Studies also report dysfunction in the temporoparietal junction and the oculomotor network that also were related to clinical parameters. We discuss these findings based on recent research on thalamocortical networks and brain stem white matter tracts.

**Summary:**

The hypothesis of frontotemporal and thalamic involvement in KLS was confirmed, and additional findings in the temporoparietal junction and the oculomotor system suggest a broader network involvement, which can be investigated by future high-resolution and multimodal imaging.

## Introduction

The Kleine-Levin syndrome (KLS) or periodic idiopathic hypersomnia is a puzzling disorder when it comes to both symptomatology and aetiology, and consequently also treatment. The cardinal KLS symptom is recurrent hypersomnia, with sleep episodes that can last as long as 2 weeks and recur several times a year. During sleep episodes, the patients are also troubled with one or more cognitive (such as language or memory impairment), psychiatric (such as derealization, apathy, depression), or behavioural dysfunctions (such as hyperphagia, hypersexuality, irritability, aggression) [1]. Between sleep periods, the patients normally are asymptomatic, except for reduced processing speed and verbal memory [2], including working memory deficits [3, 4]. Except from a few case studies, structural neuroimaging normally reports absence of pathology [5].

Over the years, several functional neuroimaging approaches have been applied to elucidate KLS aetiology and possibly assist in the diagnosis [5]. One frequently reported finding from these imaging studies is frontotemporal hypoperfusion that also persists during asymptomatic periods [6]. Another main finding is related to abnormal function in the thalamus most frequently manifested as hypoperfusion during sleep episodes [6] and increased blood-oxygen-level-dependent (BOLD) responses when patients are challenged by effortful working memory tasks [4]. However, neuroimaging findings sometimes show inconsistencies that might be related to small sample sizes, the specific phase of KLS episode at the time of neuroimaging, or the selected neuroimaging method. The most common functional neuroimaging methods in KLS research are single-photon emission computed tomography (SPECT) that measures cerebral perfusion, 18F-fluorodeoxy glucose positron emission tomography (FDG-PET) measuring glucose metabolism and functional magnetic resonance imaging (fMRI) measuring brain activation through the BOLD response to neural activity (Box 1). Although these different measures are related to each other through the coupling between neural activation, cerebral blood flow and metabolism, the different neuroimaging methods are focused on separate aspects of brain function.

Box 1 Neuroimaging methods and what they measure

**Table:** 

Here, we give a brief overview of neuroimaging methods relevant for KLS research and explain the structural, neurovascular and metabolic sources they measure.	
MRI	
MRI is the state-of-the-art method when it comes to high-resolution structural imaging of the brain. As MRI is sensitive to the interaction between protons, predominantly found in water, and different tissue compartments it gives images with excellent contrast between white and grey matter and cerebrospinal fluid, without adding external contrast agents. By adjusting MRI scanner settings, it is possible to enhance certain tissue contrasts. For example, T2-weighted (T2W) and diffusion-weighted (DWI) is often used for pathology visualisation. Diffusion tensor imaging (DTI), or tractography, is used for visualisation of white matter tracts through the enhanced diffusion of water along axons.	
fMRI	
By fMRI, it is possible to visualise the brain at work. When neurons are active, they induce release of vasoactive substances in predominantly astrocytes that triggers cerebral blood flow increase, which leads to transportation of oxygenated blood into the active area. Since the fMRI signal is sensitive to blood oxygenation through the BOLD response, fMRI provides indirect images of brain activation. fMRI is applied in two modes: (1) task-based fMRI which show brain areas that are activated by a specific task, e.g.*,* working memory tasks that have been applied in KLS research and (2) resting-state (rs) fMRI which show brain areas that are functionally connected to each other during rest, i.e.*,* functional connectivity. By similar methods, functional connectivity can also be studied during task performance. When referring to fMRI studies in this review, we use the term *BOLD response* for task-based fMRI results and *functional connectivity* for rs-fMRI results.	
Magnetic resonance spectroscopy (MRS)	
MRS is most commonly focused on proton containing substances other than water and fat (1H-MRS) but also other magnetic nuclei, for example phosphorus (31P-MRS), are possible to capture. 1H-MRS gives information of brain metabolites in the form of a spectrum where the spectral peaks are related to *concentrations* of different metabolites, such as n-acetylaspartate (NAA), and the neurotransmitters glutamate and GABA. MRS is mostly applied using a single voxel technique, where a spectrum is captured in one selected region of the brain.	
SPECT	
SPECT is a nuclear medicine imaging method that uses gamma-ray emitting radionuclides to estimate tissue function. The most common radionuclide for brain imaging is a metastable isotope of technetium, ^99m^Tc. When ^99m^Tc is bound to a certain ligand and injected to the blood stream, it passes the blood-brain barrier. When the gamma rays are captured in the scanner, they give information about cerebral *perfusion*.	
PET	
PET is another nuclear medicine imaging method that captures information from pairs of gamma rays derived from protons emitted from certain radionuclides, so-called tracers. Several PET tracers have been developed to gain information about specific neuroreceptors. However, the most common method is FDG-PET where the measured concentration of the distributed tracer corresponds to regional *glucose metabolism.*	

The aim of the current review is to report the most recent literature on neuroimaging in KLS. We specifically aimed to investigate if frontotemporal and thalamic dysfunction are key KLS signatures, and if recent research indicates other brain networks of interest that could elucidate KLS symptomatology and aetiology.

## Literature Search

In order to obtain an update of recent literature on neuroimaging in KLS, we searched for articles in Web of Science and PubMed using the keywords (Kleine-Levin syndrome or KLS) and (PET or SPECT or MRI or fMRI or MRS or DTI or neuroimaging) during the period 2013–2018. We excluded review articles, articles without neuroimaging data, and articles that were not specifically reporting KLS findings, e.g.*,* reporting findings in idiopathic hypersomnia. We also excluded clinically related articles only reporting structural MRI or CT without pathological findings.

## Recent Neuroimaging Findings in KLS

**Fig. 1:**
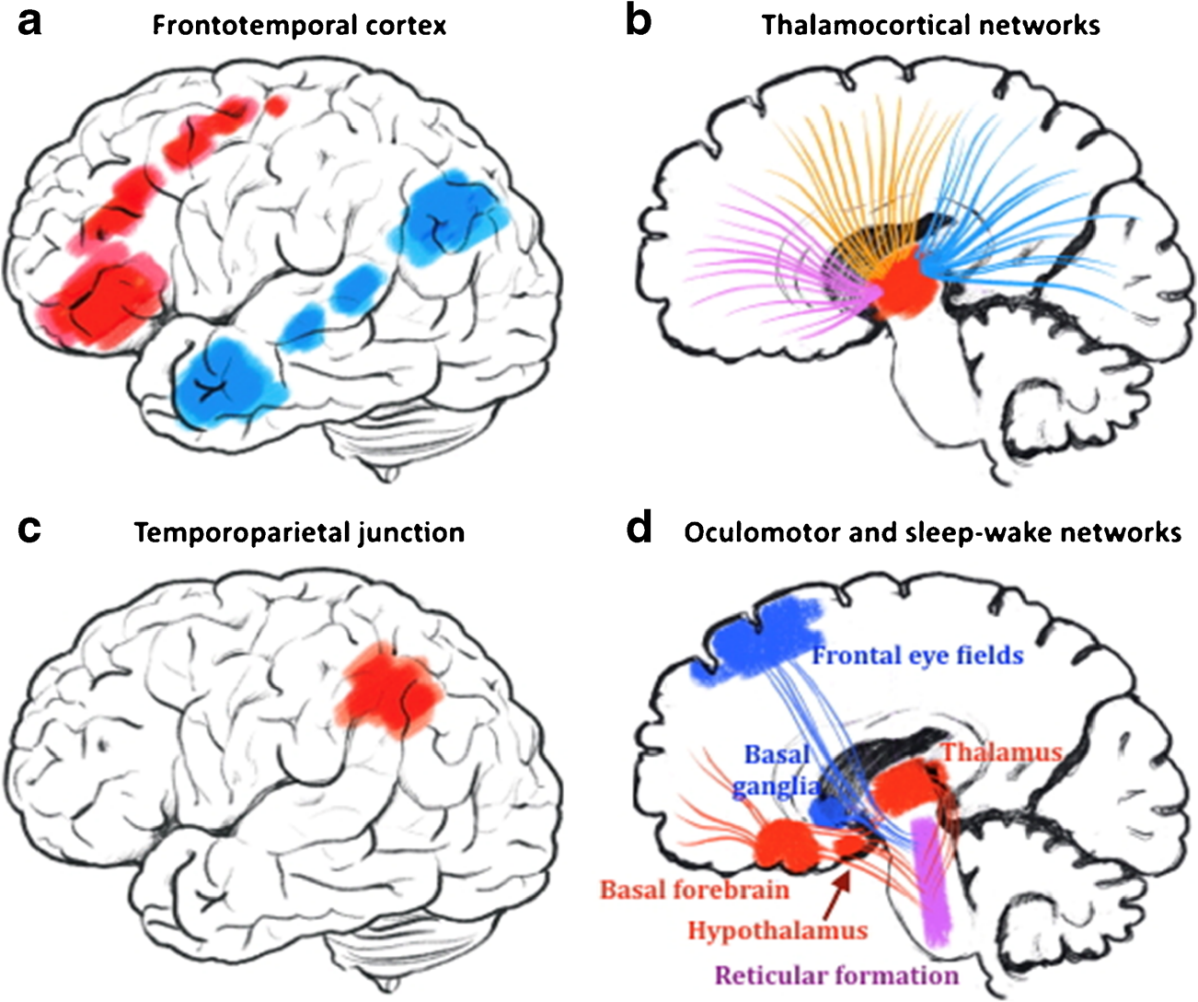
Schematic overview of suggested brain regions and networks involved in KLS according to recent neuroimaging reviews. **a** Frontotemporal regions with observed hypoperfusion and glucose hypermetabolism in KLS. **b** Thalamocortical networks with reported dysfunction in SPECT, PET, and fMRI studies. **c** The temporoparietal junction where cerebral perfusion is related to experiences of depersonalization and derealization in KLS. **d** Oculomotor and sleep-wake networks. Functional connectivity and perfusion studies show deviant function in the oculomotor network (blue) that involves nuclei in the brain stem reticular formation (purple) partially overlapping with the sleep-wake network (red)

In the literature search, we found 12 original articles on neuroimaging in KLS published 2013–2018, whereof five articles were case reports [7, 8, 9••, 10–18] (Table 1). Most studies report deviant function in the form of hypoperfusion, glucose hypermetabolism, or increased BOLD responses in frontotemporal areas (Fig. 1a) and/or the thalamus (Fig. 1b). Some studies also report additional findings in cortical and subcortical areas, whereof findings in the temporoparietal junction (Fig. 1c) and the brain’s oculomotor network (Fig. 1d) will be presented and discussed below.

**Table 1:** Summary of neuroimaging findings in the Kleine-Levin syndrome (KLS). The table lists all neuroimaging studies reported in the period 2013–2017. DWI = diffusion-weighted imaging, FDG-PET = 18-F-fluorodeoxy glucose positron emission tomography, fMRI = functional magnetic resonance imaging, MRS = magnetic resonance spectroscopy, rs-fMRI = resting-state fMRI, SPECT = single photon emission computed tomography, T2W MRI = T2-weigthed magnetic resonance imaging, NAA = n-acetylaspartate

Modality	Subjects	KLS episode	Main findings	Citation
DWI, T2W MRI	1 KLS	Prior to diagnosis	Reversible reduced diffusion in the corpus callosum splenum after encephalitis. Mild hyperintensity on T2W.	Takayanagi et al.*,* 2017 [7]
FDG-PET	1 KLS	Symptomatic and asymptomatic	Symptomatic increased glucose metabolism in bilateral thalami, caudate nuclei and lenticular nuclei	Drouet et al.*,* 2017 [8]
rs-fMRI	12 KLS, 14 HC	Asymptomatic	Reduced functional connectivity between dorsal pons and frontal eye fields. No difference in thalamic connectivity.	Engström et al.*,* 2016 [9••]
FDG-PET	1 KLS	Symptomatic	Decreased glucose metabolism in bilateral thalami.	Xie et al.*,* 2016 [10]
SPECT	41 KLS, 15 HC	Symptomatic and asymptomatic	General hypoperfusion in hypothalamus, thalamus, caudate, and anterior cingulate, orbito-frontal and temporal cortices. Symptomatic: additional hypoperfusion in right dorsomedial prefrontal cortex and right parieto-temporal junction. Depersonalization/derealization correlated with parieto-temporal hypoperfusion	Kas et al.*,* 2014 [11]
FDG-PET	4 KLS, 15 HC	Symptomatic and asymptomatic	Symptomatic increased glucose metabolism in paracentral and postcentral areas, supplementary motor area, medial frontal gyrus, thalamus and putamen. Decreased metabolism in occipital and temporal gyri. Asymptomatic KLS showed wide spread hypermetabolism compared to HC.	Dauvilliers et al.*,* 2014 [12]
fMRI	18 KLS, 26 HC	Asymptomatic	Reversed relation between thalamic activation and working memory capacity in KLS compared to HC	Engström et al.*,* 2014 [13]
rs-fMRI	1 KLS, 14 HC	Symptomatic and asymptomatic	Symptomatic reduction in functional connectivity between thalamus and dorsal pons. Asymptomatic normal thalamic connectivity	Engström et al.*,* 2014 [14]
SPECT	24 KLS	Asymptomatic	Temporal or fronto-temporal hypoperfusion	Vigren et al.*,* 2014 [15]
fMRI	18 KLS, 26 HC	Asymptomatic	Increased activation in e.g. left frontal cortex and thalamus. Increased functional connectivity between the executive and salience network and regions outside respective network.^a^	Engström et al.*,* 2013 [16]
fMRI, MRS	14 KLS, 15 HC	Asymptomatic	Inverse correlation between thalamic activation and NAA-concentration	Vigren et al.*,* 2013 [17]
MRI	1 KLS	–	Whole brain atrophy	Shi et al.*,* 2013 [18]

^a^The main scope of the study was to investigate effort-related brain activation in healthy participants and patients with working memory deficits

### Frontotemporal Cortex

A large SPECT study by Kas et al. included 41 asymptomatic KLS patients, whereof 11 also were scanned during symptomatic periods, and 15 healthy controls [11]. In line with previous single case studies [3, 19], they found frontotemporal hypoperfusion in KLS patients compared to healthy controls. In this larger case-control study, the authors specified frontal hypoperfused areas in the orbitofrontal and the anterior cingulate cortices. During symptomatic episodes, KLS patients had additional hypoperfusion in the right dorsomedial prefrontal cortex and the right temporoparietal junction. These two areas were more affected during the asymptomatic periods in patients with longer episode duration. Dauvilliers et al. studied glucose metabolism with FDG-PET and found wide spread hypermetabolism in frontotemporal cortices, as well as in the posterior cingulate cortex and the precuneus, when comparing asymptomatic KLS patients with healthy controls [12]. During sleep episodes, the difference in glucose metabolism between KLS patients and healthy controls was further pronounced involving also the inferior parietal cortex and the left insula. During the asymptomatic period, Dauvilliers and co-workers also observed areas of hypometabolism, especially in the occipital lobe. An fMRI study by Engström et al. compared 18 asymptomatic KLS patients with 26 healthy controls and observed that KLS patients had increased BOLD responses in the left frontal gyrus during effortful working memory performance [16]. They also found that KLS patients had increased functional connectivity between both the executive frontoparietal network and the salience network (involving the anterior insular and the anterior cingulate cortices) and regions outside respective network, indicating a network mix-up in KLS. That is to say, the executive and salience networks were not clearly delineated in KLS patients as they were in healthy controls.

The above studies investigated perfusion, glucose metabolism, BOLD responses and functional connectivity on a group-level comparing asymptomatic KLS patients with healthy controls or KLS patients in symptomatic vs. asymptomatic episodes. Results show statistically significant group- or state-dependent differences in frontotemporal and also parietal regions. However, what does these results on group level say about the status of individual patients? Can we use these neuroimaging methods for clinical patient assessment and diagnosis? A SPECT study by Vigren et al. reported temporal or frontotemporal hypoperfusion in 48% of 24 KLS patients investigated during their asymptomatic period or after remission [15]. This high prevalence of frontotemporal hypoperfusion in KLS suggests that SPECT perfusion could be an additive diagnostic tool.

In summary, three different neuroimaging methods show functional abnormalities in frontotemporal, and sometimes also in parietal regions, that are persistent during asymptomatic episodes suggesting involvement of primarily language, but also executive and salience networks, in KLS. This conclusion is supported by the reported deficiencies in verbal memory, both episodic and working memory [2, 13] and clinical symptoms of speech and reading impairments [20].

### Thalamus

Other key findings in the KLS neuroimaging literature is thalamic dysfunction in the form of increased BOLD responses [13, 16], hypoperfusion [11], or glucose hypermetabolism [8, 12] with abnormal patterns often extended to the striatum (Table 1). Glucose hypermetabolism is also reported to increase during symptomatic episodes [8, 12]. However, one case study reported decreased glucose metabolism in bilateral thalami [10].

An extended fMRI study in 18 asymptomatic KLS patients could reproduce findings of increased BOLD responses in the left thalamus during a verbal working memory task [16]. When making direct correlations between working memory performance and thalamic BOLD responses, it was found that lower performance in healthy subjects was associated with higher BOLD responses [13], in line with the neural efficiency hypothesis [21, 22]. Unexpectedly, a trend for the opposite pattern was found in KLS indicating that increased BOLD response in the thalamus could be a successful compensatory mechanism in high performing KLS patients.

A combined fMRI and magnetic resonance spectroscopy (MRS) study in 14 asymptomatic KLS patients and 15 healthy controls reported inverse correlation between BOLD responses and N-acetylaspartate (NAA) concentration in the thalamus [17]. NAA is a metabolite associated with neuronal concentration or neuronal health and viability. Thus, these results indicate that higher BOLD responses during working memory performance in KLS are related to thalamic neuronal loss or malfunction. A possible burnout effect in high performing KLS patients cannot be excluded since burnout and stress is related to reduce grey matter volumes [23].

Functional connectivity between the thalamus and the executive and salience networks, respectively, were increased in KLS patients when they performed an effortful working memory task [16]. During resting state, on the other hand, there was no difference in thalamic functional connectivity when comparing asymptomatic KLS patients and healthy controls [14]. However, one KLS patient that was investigated in both symptomatic and asymptomatic phases had reduced functional connectivity between the thalamus and the dorsal pons during a sleep episode [14].

In summary, thalamic malfunction continues to be a primary hypothesis in KLS aetiology as functional neuroimaging show involvement of the thalamus in the asymptomatic state with worsened dysfunction during symptomatic episodes.

### Striatum

Abnormal neuroimaging patterns in KLS are also observed in the striatum, especially in the caudate nucleus and the putamen [4, 8, 11, 12]. The striatum has been associated with several repetitive behavioural disorders such as Tourette’s syndrome and obsessive-compulsive disorders, reviewed in [24] and to Parkinson’s disease where some patients also are afflicted with compulsive symptoms, e.g.*,* hypersexuality, binge eating, pathological gambling, and compulsive shopping, reviewed in [25]. However, it is important to keep in mind that these symptoms, to a certain extent, may be connected to pharmacotherapy in Parkinson’s disease, especially L-DOPA substitution. Still, a wide range of behavioural symptoms, as those reported in KLS, seem to depend on the dopaminergic portion of the reward network. The ventral/limbic part of the network, including the anterior thalamic radiation, nucleus accumbens, caudate nucleus and the putamen, has been reported in connection with obesity, hypersexuality, aggressive behaviour and gambling [26–30]. In addition, the accumbo-frontal fasciculus and the anterior thalamic radiation are part of a cortico-striato-thalamo-cortical loop [29] which contain structures that also are targets for deep brain stimulation (DBS) in obsessive-compulsive and other psychiatric disorders [31, 32]. Thus, the neuroimaging findings in the striatum reported here might be related to behavioural symptoms in KLS; however, no scientific evidence of the relation between odd behaviour in KLS and dysfunction in the striatum has been reported as of yet. In addition, frontotemporal dysfunction may also contribute to behavioural symptoms in KLS, due to the relation between frontotemporal areas and the brain’s social control system [33].

### Temporoparietal Junction

A significant finding in the study by Kas et al. [11] was hypoperfusion in the temporoparietal junction during symptomatic episodes that correlated with experiences of depersonalization and derealization. The temporoparietal junction, as discussed by the authors, is related to the ability to perceive an embodied self [34, 35]. Interestingly, lesion and neuroimaging studies as well as studies using experimental manipulations have repeatedly associated the temporoparietal junction with so-called *out of body experiences* [36–39]*.*

### Oculomotor Network

One resting-state fMRI study including 12 asymptomatic KLS patients and 14 healthy controls reports that KLS patients have reduced functional connectivity between the dorsal pons and the frontal eye field in Brodmann area (BA) 8 [9••]. Kas et al. report decreased perfusion in BA 8 in symptomatic KLS patients and inverse correlation between perfusion in this area and mean duration of the sleep episodes [11]. The frontal eye field is involved with visual attention [40] and saccadic eye movements [41], and it is thus part of the brain’s oculomotor system through its strong connections mainly to the pontine reticular formation, thalamus and the basal ganglia [42]. Interestingly, adjacent nuclei in the pontine reticular formation are involved with sleep regulation or eye movements, which for example are manifested in rapid eye movement (REM) sleep (Fig. 1d).

## Thalamic Nuclei and Thalamocortical Networks in KLS

KLS neuroimaging literature evidence broad involvement of thalamocortical networks, without findings of structural modifications in clinical neuroradiology. The thalamus is a primary subcortical hub having a crucial modulatory role in facilitating cortical arousal, information transmission and consciousness [43]. There is strong evidence that the thalamus plays a critical role in both sleep and anaesthesia-induced unconsciousness with a consequent change in regional metabolism [44, 45]. Changes in thalamic activity can, indeed, result in altered cortical and thalamocortical oscillations or dysrhythmia [46, 47]. Its “gate-like” position for most incoming sensory information, the arousal system, and the coordination of cortical communication and computation [48, 49] is supported by a rich and complex white matter connectivity with both reciprocal and not reciprocal pathways with surrounding structures. Using diffusion tensor imaging (DTI) for tractography and fMRI for functional connectivity in larger cohorts, several authors were able to segment structural and/or functional connectivity of the thalamus by its core networks and nuclei [50•, 51•, 52•].

Interestingly, areas with increased BOLD responses or increased functional connectivity in KLS patients [13, 16] involve specific groups of thalamic nuclei. During working memory performance, KLS patients have increased BOLD responses in the left anterior and mediodorsal thalamus. The anterior thalamus is structurally and functionally connected to widespread cortical areas such as the hippocampus and cingulate cortex with proposed roles in head direction, spatial navigation and learning [53–56]. The medial dorsal thalamus is, on the other hand, believed to maintain and modulate working memory and attention/wakefulness [57, 58••] through projections to the frontal lobe and the cingulate cortex via the anterior and superior thalamic radiation [59]. Both anterior and medial dorsal thalamus play a role in saccadic eye movements through their specific projections to the frontal eye field [54–56, 60]. The pulvinar, which is involved in attention and visual salience [61, 62], is more connected to the executive and salience network in KLS during effortful working memory tasks possibly through the posterior thalamic radiation. Thus, divergent cortical function as revealed by functional neuroimaging in KLS seem in agreement with the involvement of segregated thalamocortical connectivity [50•, 51•, 52•, 63, among others].

## The Brain Stem and Sleep-Wakefulness Networks in KLS

Another key region possibly involved in KLS-affected networks is the brain stem. Arousal and sleep promoting nuclei are located in the pontine reticular formation, e.g.*,* locus coeruleus, raphe nucleus, and the pedunculopontine nuclei, and the mesencephalon-tegmental area with projections to the thalamus, hypothalamus and the basal forebrain (Fig. 1d) [58••, 64, 65]. A direct and possibly reciprocal communication between pontine nuclei (via mesencephalon) and the frontal, temporoparieto and occipital cortices are provided by the fronto-pontine tract and the temporo-parietal-occipital pontine tract, respectively. Both systems follow the fibres of the internal capsule and end into the ventral portion of the pons [66]. As a clearly distinct pathway, the cortico-spinal tract connects primary motor, supplementary motor cortex, and the parietal lobe with the medulla, but along the way through the brain stem, its fibres are intermingled with the mesencephalic and pontine nuclei. Two major white matter bundles instead follow an ascending route to the thalamus: the medial lemniscus and the spino-thalamic tract. Both bundles provide sensory information from the periphery and in their ascending path they meet several pontine and mesencephalic nuclei described above. Then, the bundles end into the thalamus where high-order neurons reach the sensory cortex via the superior thalamic radiation [66, 67]. Finally, a direct/reciprocal connection between pontine and mesencephalic nuclei, tegmental area and the hypothalamus is supported by the dorsal longitudinal fasciculus [66]. This thin pathway seems to have a crucial position in the centre of the sleep-awake regulation network.

## Uncoupling of Cerebral Blood Flow and Metabolism

In healthy subjects, cerebral blood flow and metabolism are closely related, shown as regional correlation between cerebral blood flow and cerebral metabolic rate of oxygen [68] or glucose [69]. Therefore, blood flow and metabolism have been regarded as equivalent measures of brain function. However, as reviewed here, SPECT studies consequently report hypoperfusion in cortical and subcortical areas and FDG-PET studies principally report glucose hypermetabolism in similar areas of the brain suggesting uncoupling of cerebral blood flow and metabolism in KLS. This is a seemingly inconsistent finding but uncoupling of vascular and energetic cerebral responses has previously been observed during sensory stimulation [68] and also in different disease states such as unipolar depression [69], epilepsy [70] and traumatic brain injury [71]. Regional glucose hypermetabolism, as reported in KLS, has also been observed in patients with depression [72]. In patients with malignant melanoma, increased glucose metabolism was related to self-reported fatigue [73], but in patients with mild cognitive impairment (MCI), cortical glucose hypermetabolism was suggested to be protective for Alzheimer’s disease [74].

Increased BOLD responses are strongly associated with increased neural activity, cerebral blood flow and metabolism [75]. However, when baseline cerebral blood flow is reduced, for example during hypocapnia [76] or caffeine intake [77], the BOLD response is increased, and conversely when baseline blood flow is higher than normal [78]. Therefore, hypoperfusion, a sign of reduced baseline cerebral blood flow, could lead to increased BOLD responses in KLS without concomitant increases in neural activity.

## Clinical Observations Related to Neuroimaging Findings in KLS

Our clinical observations, i.e.*,* recurrent reports from the patients and their family on visual disturbances of different kinds, are in line with the recent scientific results on reduced functional connectivity between the pons and the frontal eye fields. We have previously reported reduced working memory in several publications [3, 4, 13, 16, 17]. One of these studies [3] as well as preliminary data (Ulrici and Landtblom, unpublished data) suggests a predominant engagement of the visual working memory. Ongoing studies will hopefully elucidate this hypothesis. There are repeated reports on patients that have difficulties to interpret visual information. A striking example is the inability of KLS patients to recognise the face when looking into the mirror, as described by four of our patients. This perceptual disturbance resembles a form of temporary “ictal” proposoagnosia. Besides from this, we know from the families of almost all our cases, that the look immediately turns “empty” when the patient gets ill. Finally, we have encountered two examples of “ictal” nystagmus. Of interest is also the symptom of derealisation, the feeling that the perception is “unreal,” shown to be associated with hypoperfusion in the associative temporoparietal cortex [11, 79••].

Since the symptoms and also episode duration and frequency differ between KLS patients, it is important in future neuroimaging studies to control for these clinical observations. As reviewed here, deviant brain function during the asymptomatic phase is worsened during sleep episodes. These observations lead to the question if the deviant findings during asymptomatic periods are reminiscent effects of brain abnormalities that occur during sleep episodes. Or are the underlying abnormalities observed during the asymptomatic period causing later sleep period onset?

## Conclusions

Functional neuroimaging in KLS have evidenced involvement of frontotemporal, thalamocortical and brain stem networks. However, more detailed structural and functional analysis of the communication between the thalamus and cortical and subcortical structures that regulate sleep-wakefulness seems of primary importance. High-resolution diffusion tensor imaging (DTI) of white matter tracts is an obvious alternative to the functional neuroimaging methods reviewed here. Further, multimodal functional imaging is probably necessary to inquire into the seeming uncoupling of cerebral blood flow and metabolism in KLS, as well as to delineate vascular, metabolic and neural contributions to KLS pathology.
